# Supplementary material for: Associations of Broader Parental Factors with Children’s Happiness and Weight Status through Child Food Intake, Physical Activity, and Screen Time: A Longitudinal Modeling Analysis of South Korean Families
Source: Int J Environ Res Public Health. 2024 Feb 3;21(2):176. doi: 10.3390/ijerph21020176 (PMC10888306; doi:10.3390/ijerph21020176)
Supplement: Supplementary file 1 [file ijerph-21-00176-s001.zip › ijerph-2776153-supplementary.pdf]

Supplementary S1. Correlations among the Variables.

| | 1 | 2 | 3 | 4 | 5 | 6 | 7 | 8 | 9 | 10 | 11 | 12 | 13 | 14 | 15 | 16 | 17 | 18 | 19 | 20 | 21 | 22 | 23 | 24 | 25 | 26 | 27 | 28 | 29 |
|---|---|---|---|---|---|---|---|---|---|---|---|---|---|---|---|---|---|---|---|---|---|---|---|---|---|---|---|---|---|
| 2 | .87 | 1 | | | | | | | | | | | | | | | | | | | | | | | | | | | |
| 3 | .73 | .74 | 1 | | | | | | | | | | | | | | | | | | | | | | | | | | |
| 4 | .44 | .43 | .33 | 1 | | | | | | | | | | | | | | | | | | | | | | | | | |
| 5 | .43 | .42 | .33 | .88 | 1 | | | | | | | | | | | | | | | | | | | | | | | | |
| 6 | .35 | .36 | .29 | .72 | .74 | 1 | | | | | | | | | | | | | | | | | | | | | | | |
| 7 | -.43 | -.43 | -.43 | -.24 | -.24 | -.22 | 1 | | | | | | | | | | | | | | | | | | | | | | |
| 8 | -.42 | -.43 | -.43 | -.23 | -.23 | -.22 | .84 | 1 | | | | | | | | | | | | | | | | | | | | | |
| 9 | -.41 | -.41 | -.41 | -.23 | -.21 | -.19 | .66 | .68 | 1 | | | | | | | | | | | | | | | | | | | | |
| 10 | -.29 | -.30 | -.22 | -.47 | -.44 | -.43 | .30 | .30 | .28 | 1 | | | | | | | | | | | | | | | | | | | |
| 11 | -.30 | -.31 | -.23 | -.49 | -.47 | -.43 | .31 | .32 | .30 | .80 | 1 | | | | | | | | | | | | | | | | | | |
| 12 | -.28 | -.29 | -.23 | -.42 | -.41 | -.39 | .29 | .27 | .31 | .64 | .65 | 1 | | | | | | | | | | | | | | | | | |
| 13 | .17 | .17 | .18 | .13 | .12 | .13 | -.18 | -.21 | -.19 | -.11 | -.13 | -.10 | 1 | | | | | | | | | | | | | | | | |
| 14 | .18 | .18 | .19 | .11 | .11 | .12 | -.13 | -.16 | -.15 | -.09 | -.08 | -.09 | .41 | 1 | | | | | | | | | | | | | | | |
| 15 | .17 | .19 | .16 | .13 | .11 | .12 | -.18 | -.19 | -.17 | -.13 | -.12 | -.12 | .45 | .50 | 1 | | | | | | | | | | | | | | |
| 16 | .13 | .14 | .15 | .10 | .10 | .11 | -.15 | -.17 | -.13 | -.07 | -.06 | -.06 | .45 | .55 | .50 | 1 | | | | | | | | | | | | | |
| 17 | .16 | .16 | .14 | .21 | .20 | .19 | -.13 | -.14 | -.11 | -.18 | -.19 | -.13 | .19 | .17 | .20 | .21 | 1 | | | | | | | | | | | | |
| 18 | .13 | .14 | .12 | .21 | .21 | .18 | -.14 | -.13 | -.12 | -.17 | -.17 | -.16 | .08 | .22 | .18 | .16 | .50 | 1 | | | | | | | | | | | |
| 19 | .14 | .17 | .12 | .22 | .24 | .23 | -.16 | -.15 | -.11 | -.21 | -.21 | -.19 | .12 | .14 | .20 | .17 | .53 | .56 | 1 | | | | | | | | | | |
| 20 | .14 | .15 | .12 | .20 | .19 | .19 | -.12 | -.12 | -.10 | -.14 | -.15 | -.09 | .08 | .16 | .15 | .21 | .54 | .60 | .55 | 1 | | | | | | | | | |
| 21 | .11 | .12 | .09 | .09 | .08 | .05 | -.17 | -.19 | -.14 | -.07 | -.09 | -.09 | .14 | .08 | .14 | .08 | .03 | .07 | .09 | .03 | 1 | | | | | | | | |
| 22 | -.02 | -.02 | -.03 | .04 | .02 | .02 | .02 | .01 | .04 | -.01 | .01 | -.01 | -.03 | -.01 | -.03 | -.04 | -.03 | .03 | -.01 | .00 | .14 | 1 | | | | | | | |
| 23 | .00 | -.01 | -.02 | .01 | .05 | .05 | -.03 | -.04 | -.01 | -.03 | -.04 | .00 | .08 | .07 | .08 | .10 | .03 | .06 | .06 | .05 | .09 | .06 | 1 | | | | | | |
| 24 | -.20 | -.22 | -.14 | -.16 | -.16 | -.10 | .13 | .13 | .14 | .13 | .12 | .09 | -.11 | -.09 | -.11 | -.04 | -.09 | -.07 | -.10 | -.06 | -.12 | .09 | -.05 | 1 | | | | | |
| 25 | .00 | .00 | .02 | .00 | -.03 | .01 | .00 | .01 | .04 | .04 | .06 | .00 | .00 | .01 | -.03 | .00 | -.05 | -.08 | -.09 | -.02 | .01 | .04 | -.01 | .11 | 1 | | | | |
| 26 | .03 | .02 | .03 | .02 | .00 | .00 | .00 | .00 | .04 | .01 | .05 | -.02 | .00 | .00 | .00 | .03 | -.08 | -.09 | -.08 | -.03 | .03 | .06 | .03 | .14 | .88 | 1 | | | |
| 27 | -.01 | -.02 | -.01 | -.01 | -.03 | -.01 | .00 | .01 | .06 | .05 | .07 | .02 | -.02 | -.02 | -.02 | .00 | -.08 | -.11 | -.08 | -.04 | .02 | .06 | .05 | .14 | .83 | .90 | 1 | | |
| 28 | .05 | .05 | .05 | .05 | .05 | .04 | -.04 | -.07 | -.05 | -.07 | -.06 | -.05 | .06 | .04 | .08 | -.01 | .06 | .08 | .06 | .03 | .02 | -.01 | -.02 | -.13 | -.03 | -.01 | -.02 | 1 | |
| 29 | .09 | .10 | .09 | .05 | .04 | .04 | -.11 | -.10 | -.12 | -.08 | -.08 | -.07 | .06 | .02 | .04 | .00 | .04 | .05 | .07 | .01 | .07 | -.04 | -.02 | -.18 | -.05 | -.05 | -.05 | .34 | 1 |
| 30 | .10 | .10 | .09 | .09 | .08 | .06 | -.11 | -.11 | -.13 | -.10 | -.08 | -.07 | .06 | .02 | .06 | .01 | .05 | .02 | .03 | .01 | .05 | .00 | .00 | -.16 | -.04 | -.05 | -.06 | .26 | .45 |

Note. Red indicates a significant positive correlation. Blue indicates a significant negative correlation.

[1] Mother's happiness - In general, I consider myself happy;
[2] Mother's happiness - Compared with most of my peers, I consider myself happy;
[3] Mother's happiness - I enjoy life regardless of what is going on;
[4] Father's happiness - In general, I consider myself happy;
[5] Father's happiness - Compared with most of my peers, I consider myself happy;
[6] Father's happiness - I enjoy life regardless of what is going on;
[7] Mother's parenting stress – I am not sure if I could be a good parent;
[8] Mother's parenting stress – I am not confident that I can raise my child properly;
[9] Mother's parenting stress – I feel my child is behind his/her peers because I do not parent him/her well;
[10] Father's parenting stress – I am not sure if I could be a good parent;
[11] Father's parenting stress – I am not confident that I can raise my child properly;
[12] Father's parenting stress – I feel my child is behind his/her peers because I do not parent him/her well;
[13] Mother's play engagement – I do arts and crafts with my child;
[14] Mother's play engagement – My child and I play board games and do puzzles together;

[15] Mother's play engagement - My child and I talk about nature and do science projects together;
[16] Mothe's play engagement - My child and I build something or play with toys together;
[17] Father's play engagement – I do arts and crafts with my child;
[18] Father's play engagement – My child and I play board games and do puzzles together;
[19] Father's play engagement - My child and I talk about nature and do science projects together;
[20] Father's play engagement - My child and I build something or play with toys together;
[21] Child healthy eating;
[22] Child unhealthy eating;
[23] Child physical activities;
[24] Child screen time;
[25] Child weight status at age 7;
[26] Child weight status at age 8;
[27] Child weight status at age 9;
[28] Child happiness at age 7;
[29] Child happiness at age 8;
[30] Child happiness at age 9.
*$p < .05$
